# Dormancy Season Is Key to Submergence Tolerance of Annual Plant Seeds in the Drawdown Zone of the Three Gorges Reservoir

**DOI:** 10.3390/plants15111626

**Published:** 2026-05-26

**Authors:** Feng Lin, Qiaoli Ayi, Minjia Ge, Tianjiang Liu, Jiahao Luo, Xinxin Tian, Yingxi Xu, Hongjingzheng Jiang, Songping Liu, Xiaoping Zhang, Bo Zeng

**Affiliations:** 1 Key Laboratory of Eco-Environments in Three Gorges Reservoir Region (Ministry of Education), Chongqing Key Laboratory of Plant Ecology and Resources in Three Gorges Reservoir Region, School of Life Sciences, Southwest University, Beibei, Chongqing 400715, China; 2 Jiangxi Province Key Laboratory of Wetland Plant Resources Conservation and Utilization, Lushan Botanical Garden, Jiangxi Province and Chinese Academy of Sciences, Jiujiang 332900, China

**Keywords:** anti-seasonal flooding, environmental filter, seed germination, phylogenetic signal, seed bank, seed dormancy, water level fluctuation

## Abstract

Large reservoir construction generates vast drawdown zones characterized by novel hydrological regimes that impose unprecedented selective pressures. While annual plants serve as pioneer colonists during secondary succession in these ecosystems, the mechanisms allowing their seeds to persist through prolonged anti-seasonal flooding remain poorly understood. We investigated how seed germination responses to extreme submergence are influenced by dormancy traits and phylogenetic history. We conducted a field experiment on 44 common annual plant species in the Three Gorges Reservoir drawdown zone. Seeds were subjected to maximum submergence depths of 0 m (control), 5 m, 10 m, 15 m, and 20 m, along the reservoir’s hydrological gradient. Post-submergence germination percentages were measured and analyzed using linear and Bayesian phylogenetic mixed-effects models, with seed dormancy status, seed type, season, and species’ phylogenetic relationships as explanatory variables. Submergence significantly reduced overall seed germination (*p* < 0.001), but more than 75% of species retained germination capacity even after 20 m of submergence. Germination percentage distributions shifted from near-normal to bimodal with increasing depth. Although the regression of squared PIC values against phylogenetic branch lengths showed a significant relationship, phylogenetic signal for germination percentages was weak and non-significant across all depths (Pagel’s λ < 0.101, Blomberg’s K < 0.228, *p* > 0.05). Bayesian models revealed that dormancy season significantly interacted with submergence depth (Estimate = −1.41, 95% CrI [−2.16, −0.67]). Seeds dormant during autumn-winter maintained stable germination percentages across depths, while germination of spring-summer dormant seeds declined significantly with increasing depth. Our findings demonstrate that annual plant seeds possess widespread, species-specific tolerance to extreme submergence. This tolerance is primarily driven by environmental filtering rather than phylogenetic history. The seasonality of dormancy is a crucial adaptive mechanism, enabling seeds, particularly those dormant in autumn-winter, to withstand the harsh conditions of the Three Gorges Reservoir drawdown zone. This study provides a functional trait-based framework for selecting suitable species for the ecological restoration of reservoir drawdown zones globally.

## 1. Introduction

The operation of large dams creates massive drawdown zones—areas periodically inundated and exposed due to water-level fluctuations [1,2]. These zones are critical yet vulnerable ecosystems. In the initial stages of reservoir operation, primary vegetation is severely damaged [3,4]. Annual plants become the dominant life-form and pioneer group during the early secondary succession in these drawdown zones [5,6,7], playing a vital role in stabilizing shorelines, preventing erosion, and enhancing ecosystem services. As the principal regeneration mechanism in disturbance-prone ecosystems, seed germination exhibits acute sensitivity to hydrological fluctuations characteristic of reservoir drawdown zones [8,9]. For annual plants dominating these habitats, post-submergence germination plasticity constitutes the pivotal bottleneck determining recruitment success [10,11]. Nevertheless, dam-regulated inundation regimes generate distinct hydraulic stressors—notably anti-seasonal flooding pulses—that remain experimentally underexplored concerning transient seed banks. Specifically, knowledge gaps persist regarding how the fate and germination of seeds in riparian annuals respond to submergence due to dam regulation.

Hydrological regime, particularly the timing (season) and depth of flooding, is a master environmental filter determining plant survival in riparian zones [12,13]. Hydrological regime acts as a master environmental filter through two synergistic pathways: (i) temporal displacement, wherein winter flooding disrupts typical autumn germination windows via hypoxic suppression of metabolic reactivation [14], and (ii) depth-stratified mortality where prolonged submergence induces irreversible imbibition damage to embryonic tissues [15]. In dam-regulated drawdown zones, such filtering manifests most acutely in annual species whose entire lifecycle must synchronize with truncated non-flooded periods. It remains unclear how the germination ability of annual plant seeds responds to the gradient of submergence depth in the reservoirs of large dams.

Seed dormancy is a key evolutionary adaptation that allows plants to avoid germinating under unfavorable conditions [16]. The presence/absence of dormancy, its type, and its seasonal timing collectively dictate the germination phenology of annual plants [17,18,19,20]. These dormancy traits are supposed to be critical in mediating seed responses to the flooding regimes of drawdown zones [17,21,22]. Dormancy phenotypes constitute adaptive bet-hedging strategies against stochastic flooding events, operationalized via three tiers of control: Physiological gatekeeping, non-deep physiological dormancy imposes obligate after-ripening requirements that buffer against transient moisture cues during unstable hydroperiods [16]; Morphological constraint, physical dormancy mechanisms (e.g., impermeable seed coats) create time-delayed germination, preventing seedling emergence until receding-water phases [16,20]; Phenological alignment, seasonal dormancy cycling, fine-tunes responsiveness to photoperiod/temperature signals, ensuring synchronicity with optimal establishment windows [23]. Nevertheless, the interaction between dormancy characteristics and hydrological regime, and how they jointly influence germination success, remains unexplored.

While some plant traits show strong phylogenetic conservatism [24,25,26], the extent to which seed germination specifically under submergence exhibits such patterns remains an open question. If seed germination responses to submergence are phylogenetically constrained, we would expect closely related species to exhibit similar tolerance levels. This would allow for the prediction of suitable restoration species based on phylogeny. Conversely, while strong environmental filtering might override phylogenetic history [27,28], the specific filtering exerted by prolonged flooding directly targets key germination traits needed for establishment success during inundation windows [29]. Specifically, tolerance to oxygen deprivation (anoxia tolerance), rapid underwater coleoptile elongation, and/or precise timing of germination relative to flood recession become critical selective agents [15,22]. Such intense selection pressure could theoretically lead to two distinct outcomes regarding phylogenetic structure: (a) If these key hydrologically sensitive germination traits are deeply phylogenetically conserved within certain clades adapted to aquatic environments, then close relatives sharing those ancestral adaptations will dominate (‘niche conservatism’). (b) Alternatively, convergent evolution driven by ubiquitous selection pressures could cause disparate lineages to independently evolve functionally analogous germination syndromes capable of surviving submersion (’environmental convergence’), thereby weakening any observable phylogenetic signal. A powerful empirical test is needed to discern the role of phylogeny versus environment in shaping germination strategies in this novel ecosystem.

The anti-seasonal impoundment (winter) and discharge (summer) operation model of the Three Gorges Reservoir (TGR) has formed a drawdown zone with a 30 m vertical variation and prolonged flooding (up to 9 months; September to May), creating a stark hydrological gradient [30]. Whereas this system offers a premier natural laboratory for studying hydroperiod-seedbank interactions, critical lacunae persist regarding how phylogenetically structured functional traits mediate germination resilience under engineered anti-seasonal flooding regimes, particularly for ephemeral annuals dominating drawdown communities. To resolve this deficit, we hypothesize: (i) maximum submergence depth operates as a deterministic environmental filter on germinability; (ii) niche conservatism generates detectable phylogenetic signal in submersion tolerance thresholds; and (iii) endogenous dormancy syndromes modulate post-inundation viability outcomes. Accordingly, this study quantified germination responses across submerged depth gradients for 44 common annual species native to the TGR region, explicitly testing: (1) depth-dependent germination percentage; (2) phylogenetic signal strength; and (3) covariate effects of seed dormancy status/type/temporal triggers with submergence depth and phylogenetic background.

## 2. Results

### 2.1. Overall Germination Response to Submergence

**Figure 1:**
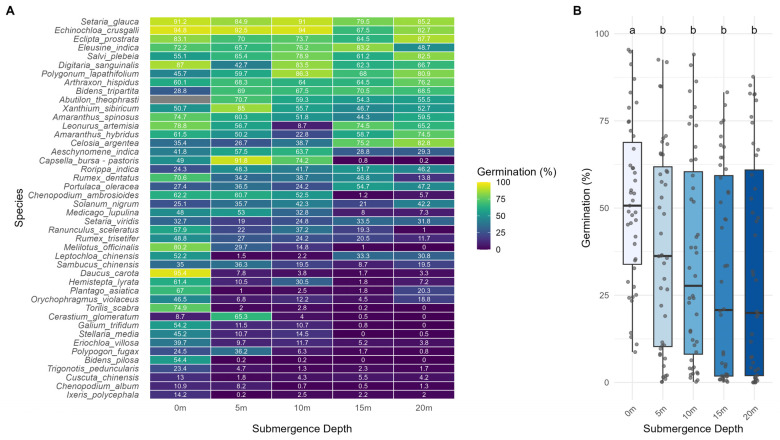
Heatmap of seed germination percentage across species after exposure to different submergence depths (**A**) and boxplots of seed germination percentages for all species (44 species) at each submergence depth post-treatment (**B**). The gray color in (**A**) of *Abutilon_theophrasti* at 0 m indicates data missing. Boxes indicate first-third quartile range (25th–75th percentile), with central line marking median values. Whiskers extend to the minimum/maximum observations. Letters a and b represent statistically distinct groupings based on post-submersion germination percentages across different inundation depths (*p* < 0.05), where identical letters denote nonsignificant differences between treatment levels.

Under non-submerged control conditions (0 m), seed germination exhibited substantial interspecific variation (range: 10–95%; mean ± SE: 51.3% ± 3.56; *n* = 44 species; Figure 1A). Submergence universally suppressed germination relative to controls (aggregate reduction: ~25–40%; *p* < 0.001; Figure 1B), yet surprisingly maintained viability thresholds—over 75% of taxa retained germinability post-20 m exposure despite increasing zero-germinator incidence with depth (species count progression: 6 → 15 from 5 m → 20 m; Figure 1A and Figure S1). Crucially, germination responses demonstrated resilience rather than determinism along the hydrostatic gradient, evidenced by nonsignificant differences among submerged treatments (mean range: 31–39%; Figure 1A and Figure S1).

Submergence imposed strong inhibitory pressures on seed germination relative to control conditions (non-submerged control: 51.95% ± 12.34 CI_95_%; Table 1). Depths ≥ 5 m universally suppressed germination (all *p* < 0.001), yet divergent trajectories emerged: maximal depression occurred at 15 m (−21.56 units, Cohen’s d = 1.17), followed by attenuated effects at 20 m (−19.58 units), indicative of biochemical compensation thresholds tied to respiratory metabolism (Table 1). Notably, species-level random effects dominated model variance (Table 1), a pattern aligned with environmental filtering theory wherein flooding regimes select for bet-hedging strategies in stochastic habitats.

### 2.2. Shift in Germination Distribution

The frequency distribution of germination percentage across species changed markedly with submergence depth (Figure S1). The distribution for the non-submerged control was approximately normal. As submergence depth increased, the distribution gradually shifted towards a bimodal pattern: one peak of species with low germination percentages (increasingly towards 0%) and another peak of species maintaining germination percentages above 50%.

### 2.3. Phylogenetic Signal and Constraints

Closely related species were not more similar in their germination response than expected by random chance (Figure S2). Phylogenetic signals for germination percentage were consistently weak and non-significant across all submergence depths (Pagel’s λ range: 0.000–0.101; Blomberg’s K range: 0.000–0.228; all *p*-values > 0.05, Figure S2).

**Figure 2:**
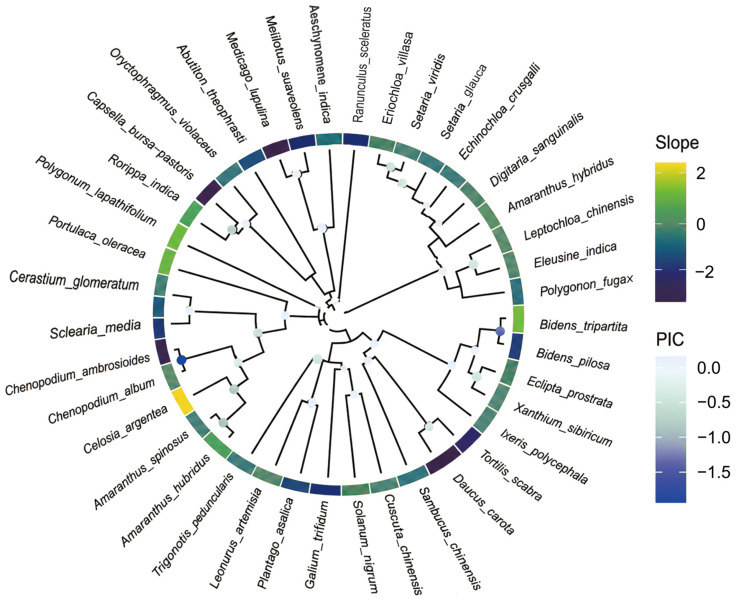
Linear regression slopes of seed germination percentage responses to different submergence depths and results of phylogenetic independent contrasts (PIC) analysis for these slopes. There are two key components: (1) the linear regression slopes (Slope, the circle heatmap) quantifying the response of seed germination percentage to varying submergence depths (0 m, 5 m, 10 m, 15 m, 20 m) for each species, reflecting the magnitude and direction of germination percentage changes with increasing submergence depth; (2) results of phylogenetic independent contrasts (PIC) analysis applied to these slopes, which accounts for phylogenetic relatedness among the 44 species to disentangle evolutionary signals from shared ancestry. PIC values indicate the phylogenetically corrected differences in response slopes between closely related species.

**Figure 3:**
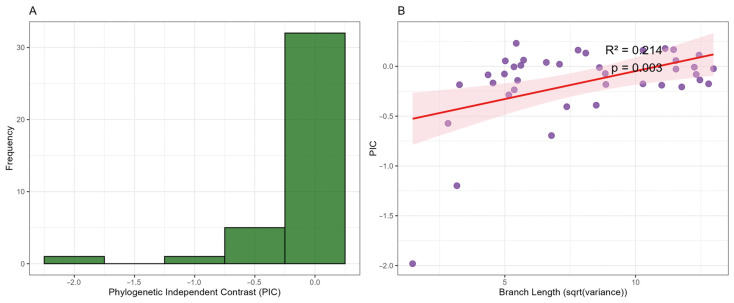
Frequency distribution of phylogenetic independent contrasts (PIC) for the slopes of linear fits describing species-specific seed germination percentage responses to different waterlogging depths (**A**), and regression analysis between these PIC values and phylogenetic branch lengths (**B**).

Germination sensitivity to submergence depth displayed exceptional interspecific divergence (slope range: −4 to 3), indicating fundamentally distinct submergence-response syndromes across taxa. Although Phylogenetic Independent Contrasts revealed moderate constraint magnitudes (PIC concentration: −2.0–0.25; Figure 2 and Figure 3A), multiple converging metrics decisively rejected phylogenetic determinism: weak evolutionary trajectory associations (branch-length regression R^2^ = 0.214, *p* = 0.003; Figure 3B) and nonsignificant phylogenetic signals under optimal modeling (λ ≈ 0, K = 0.123; Figure S1).

### 2.4. The Role of Dormancy Traits in Submergence Tolerance

Dormancy was prevalent among the studied species; 42 out of 44 species had dormant seeds at maturity, and two species were classified as non-dormant. Among the dormant species, 12 exhibited conditional dormancy, two exhibited physical dormancy, and 28 exhibited physiological dormancy. Based on fruiting period and ecology, dormancy season was classified as spring-summer for 22 species and autumn-winter for 20 species (Table S1).

**Figure 4:**
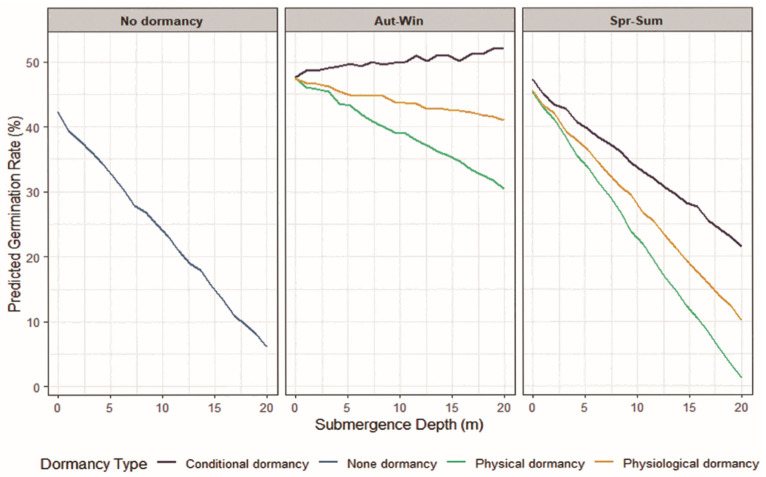
Predicted germination responses of seeds with different dormancy types and seasons to increasing submergence depth. Values are posterior mean germination percentage estimated from a Bayesian phylogenetic mixed model (brms). Predicted values were generated from the model: GerminationRate~WaterlogDepth × (Dormancy + DormancyType + DormancySeason) + (1|gr (Species, cov = phylo_cov)). Panels are faceted by dormancy season, and lines represent different dormancy types: no dormancy, conditional dormancy, physical dormancy, and physiological dormancy.

The Bayesian Phylogenetic Mixed Model revealed that dormancy season was the primary trait interacting with submergence depth to influence germination success (Table 2, Figure 4). Seeds characterized by spring-summer dormancy demonstrated heightened vulnerability to depth-dependent inhibition, evidenced by a robust negative interaction (Estimate = −1.41, 95% CrI [−2.16, −0.67]). This indicates that for seeds with spring-summer dormancy, germination percentage declined more steeply with increasing submergence depth compared to seeds dormant in autumn-winter. Specifically, seeds classified as autumn-winter dormant maintained a relatively stable germination percentage across the submergence depth gradient (Figure 4). In contrast, species with spring-summer dormancy exhibited a pronounced and significant decrease in germination as submergence depth increased. Among dormancy types, conditional dormancy appeared to confer the most stable germination performance across depths, though the interaction effects for specific dormancy types (Physical, Physiological) with depth were not statistically significant (95% CrI included zero; Table 2). Non-dormant seeds also showed a declining trend with depth.

## 3. Discussion

Our study provides a comprehensive functional-trait-based understanding of how annual plant seeds survive the extreme conditions of a mega-reservoir’s drawdown zone. The key finding is that environmental filtering acting on specific dormancy traits, rather than phylogenetic history, shapes the seedling pool in this novel ecosystem.

### 3.1. Widespread but Species-Specific Submergence Tolerance

The significant suppression of cumulative germination capacity validates submergence as a critical community assembly filter. Remarkably, >75% of studied annual species maintained germination competence following 20 m-depth submersion, indicating pervasive adaptive tolerance in drawdown zone annuals. A dichotomous response pattern emerged, with species diverging into distinct functional groups: hydro-tolerant specialists versus ruderal strategists. Such bifurcation reflects intense selective pressures imposed by the Three Gorges Reservoir’s inverted hydrological regime [14,31]. The shift from a near-normal to a bimodal distribution with increasing depth is a classic signature of environmental filtering, effectively partitioning the species pool based on tolerance thresholds.

### 3.2. The Primacy of Environmental Filtering over Phylogeny

The uniformly low phylogenetic signals across metrics (Pagel’s λ < 0.101, Blomberg’s K < 0.228; all *p* > 0.05) combined with nonsignificant PIC covariation (R^2^ = 0.001, *p* = 0.873) demonstrate limited predictive power of phylogeny for submergence-induced germination characteristics. Such decoupling between lineage history and trait expression is compatible with multiple non-exclusive processes: First, adaptive convergence under persistent hydro-edaphic filtering [26]. Second, Stochastic phenotypic diversification via genetic drift [32]. Third, phylogenetic niche conservatism operating at finer taxonomic scales is undetected here [33]. Our data align most parsimoniously with the first scenario, given known directional selection gradients in riparian zones [34,35], though explicit tests of selective regimes remain warranted. Pragmatically for restoration, functional trait screening (e.g., dormancy season) outperforms coarse phylogenetic proxies when targeting submergence-tolerant germinants.

### 3.3. Dormancy Season as a Key Adaptive Trait

The most significant finding of this study is the identification of dormancy season as the master regulator of submergence tolerance. Quantitatively validated by a strongly inhibitory interaction coefficient for SubmergenceDepth × DormancySeason: Spring-Summer (Estimate = −1.41, 95% CrI [−2.16, −0.67]), this reveals a mechanistic principle: propagules undergoing obligate dormancy during autumnal-winter intervals—synchronic with maximal reservoir impoundment—display superlative recuperative aptitude. Our findings align with the report of flood-induced dormancy cycling as a key tolerance mechanism [22], but contrast in highlighting environmental filtering over phylogenetic niche conservatism. Autumnal-winter dormancy predominance plausibly circumvents energetically inefficient germination initiation amid suboptimal submerged conditions, embodying an evolutionary avoidance paradigm. Furthermore, endogenous metabolic suspension enhances tolerance to dissolved oxygen deficits and photon flux deprivation [18,23,36,37]. Conversely, seeds dormant during spring-summer are primed to germinate as temperatures rise and water recedes. Submersion during their intended germination season creates a physiological mismatch, likely leading to premature germination underwater, energy depletion, or reduced viability upon drawdown [22,38]. Thus, the precipitously attenuated germination kinetics observed across depth gradients stem principally from chronobiological desynchronization relative to anthropogenic hydrologic pulses.

### 3.4. Implications for Ecological Restoration and Community Dynamics

Our trait-based findings have direct practical applications. The traditional approach of using phylogenetic relatedness to select species for restoration (e.g., the “phylogenetic conservatism” hope) [39,40,41] would be ineffective here, as seed submergence tolerance is not clustered phylogenetically. Instead, restoration efforts should focus on selecting species based on key functional traits, such as those possessing autumn-winter dormancy in the context of the water regime in the riparian zone of the TGR. Screening local species for this trait could rapidly identify ideal candidates for revegetation projects aimed at stabilizing banks and enhancing biodiversity in the TGR and similar mega-reservoirs worldwide.

Furthermore, our results help predict the long-term vegetation dynamics of the drawdown zone. Environmental filtering on the seed bank based on dormancy season and dormancy types is likely a major driver of community assembly. We would expect a future plant community increasingly dominated by species whose regenerative strategy (dormancy season) is pre-adapted or adapts to the anti-seasonal flooding regime. This could lead to novel communities with no historical analog.

## 4. Materials and Methods

### 4.1. Study Species and Seed Collection

Seeds of 44 annual plant species (Table S1) were selected based on their high frequency in pre-experimental vegetation surveys across representative riparian communities within the Three Gorges Reservoir area. This sampling strategy aimed to cover dominant functional groups while ensuring ecological relevance for flood disturbance studies. Mature seeds were field collected from at least 10 mother plants during peak dispersal periods in the year the experiment was conducted, followed by standardized cleaning, desiccation, and storage in room conditions with natural temperature and humidity fluctuation, using hermetic paper packaging.

### 4.2. Submergence Experiment

At the drawdown zone in the Zhong County section of TGR (106°24′57″ E, 29°19′14″ N), we established four elevation treatments (155, 160, 165, and 170 m a.s.l.) corresponding to maximum submergence depths of 20, 15, 10, and 5 m (Table 3). Five replicate seed bags (200 seeds in each seed bag) per species/elevation were deployed pre-impoundment and retrieved post-drawdown. Retrieved seeds were immediately transferred to germination beds (located at the Ecological Research Base of Southwest University, within the TGR region). The non-submerged controls were surface sown at the beginning of the submergence at 155 m in September.

### 4.3. Germination Test

Retrieved seeds were cleaned and tested for germination under outdoor seed beds for each species. Germination was monitored regularly, and a seed was considered germinated upon radicle emergence ≥ 2 mm. The final germination percentage was calculated for each replicate after 3 months with no further germination [14].

### 4.4. Dormancy Classification

Seed dormancy for each species was classified at maturity according to the scheme of Baskin and Baskin [16]. We recorded: (1) Dormancy status (Dormancy: Yes/No), (2) Dormancy type (Conditional dormancy-corresponding to the non-deep physiological dormancy in this work, here the use of “conditional dormancy” because this designation applies where partial dormancy attenuation occurred under seasonally fluctuating temperatures without obligatory scarification; Physical dormancy; Physiological dormancy- specifically, this type links to the deep physiological dormancy in this work; Non-dormant), and (3) Dormancy season (designated as either Spring-Summer or Autumn-Winter) was operationally defined through integrative analysis of seed dormancy status, fruiting phenology, and empirically verified germination characteristics. Categorization relied upon rigorous quantification of phenological synchrony between fruit dispersal phases and germination initiation thresholds under field conditions: seeds possessing confirmed dormancy mechanisms that dispersed during autumn/winter intervals yet exhibited germination primarily in spring/early summer were assigned to the Autumn-Winter class; reciprocally, dormant seeds dispersing in spring/summer periods with predominant autumn/early winter germination onset comprised the Spring-Summer category. For species listed with no dormancy (Dormancy = “no”), the dormancy type was recoded as “none” and dormancy season as “None dormancy” (Table S1).

### 4.5. Statistical Analysis

Raw germination percentage data were imported and transformed from a wide to a long format using the tidyverse suite of packages [42]. To account for phylogenetic non-independence among species, a phylogenetic tree was constructed. The species list was submitted to the V. PhyloMaker2 package using the GBOTB.extended. TPL megatree and the ‘S3’ scenario, which efficiently generates a phylogeny for any list of vascular plant species by binding them to the backbone tree [43]. The resulting phylogeny was pruned to include only species present in the germination dataset and saved in Newick format for subsequent phylogenetic related analyses.

We employed linear mixed-effects models (LMMs) to assess the fixed effects of submergence depth and its interactions with dormancy traits (Dormancy, DormancyType, DormancySeason) on germination percentage, with plant species included as a random intercept term to control for pseudoreplication. The model was fitted using the lmer function from the lme4 package with the BOBYQA optimizer [44]. The significance of fixed effects was evaluated using Satterthwaite’s method for degrees-of-freedom approximation from the lmerTest package [45]. Post hoc pairwise comparisons among submergence depths were conducted using estimated marginal means (EMMs) from the emmeans package [46], and significance groups were assigned using the multcompView package [47].

The strength of phylogenetic signal in germination percentage at each submergence depth was quantified using two metrics: Pagel’s λ and Blomberg’s K. These were calculated using the phylosig function from the phytools package [43]. A significant phylogenetic signal (*p* < 0.05) indicates that closely related species exhibit more similar germination responses to submergence than distantly related species. Phylogenetic generalized least-squares (PGLS) analysis was conducted using the λ model to allow the strength of phylogenetic signal to deviate from a pure Brownian motion expectation. The estimated λ was zero, indicating that the fixed effects (dormancy type and season) fully accounted for the phylogenetic dependence among species. Consequently, the model effectively reduced to an ordinary least-squares regression, but we nonetheless report the PGLS results to maintain analytical consistency.

We further implemented a Bayesian framework to build a more robust model that incorporated phylogenetic relatedness as a random effect. A phylogenetic covariance matrix was derived from the constructed tree using the vcv.phylo function. This matrix was integrated as a prior for the species-level random intercepts in a Bayesian mixed model fitted with the brms package [48], which provides a full probability model for uncertainty quantification. We used weakly regularizing priors (normal distribution for fixed effects, Cauchy for random effects) and ran four Markov Chain Monte Carlo (MCMC) chains for 4000 iterations each, with a warm-up of 2000 iterations. Model convergence was assessed by examining trace plots, R-hat statistics, and effective sample sizes. Posterior predictive checks were performed to evaluate the model’s fit to the observed data.

All statistical analyses and visualizations were performed using R software (version 4.5.1, R Core Team, Vienna, Austria) [49]. All figures were generated using ggplot2 and related packages [50]. A combined heatmap and boxplot visualization was created to display species-specific germination responses and overall trends across depths, with significance letters denoting post hoc (Tukey) groups. The phylogenetic tree was visualized using ggtree and annotated with PIC (Phylogenetically Independent Contrasts) values and species-specific germination slope coefficients. Conditional effects plots from the Bayesian model were used to visualize interaction effects.

## 5. Conclusions

In conclusion, our study demonstrates that the high submergence tolerance observed in many annual plant seeds in the TGR drawdown zone is a widespread but species-specific phenomenon, driven primarily by environmental filtering on a key phenological trait—seed dormancy season. The absence of phylogenetic constraints indicates convergent evolution under the strong selective pressure of the reservoir’s unique anti-seasonal hydrological regime. Autumn-winter dormancy emerges as a critical adaptive trait, allowing seeds to persist through prolonged winter inundation and successfully germinate during the summer drawdown period. These findings shift the focus from phylogeny to functional traits for understanding community assembly and provide a scientifically robust framework for selecting species for the ecological restoration of the Three Gorges Reservoir and other large reservoir drawdown zones globally.

## Figures and Tables

**Table 1:** Fixed effects of submergence depth on seed germination percentage from the Linear Mixed-effects Model.

Term	Estimate	Std. Error	df	t Value	Pr (>|t|)
(Intercept)	51.849	4.363	90.872	11.883	<2 × 10^−16^ ***
5 m	−13.274	3.943	171.13	−3.366	0.000941 ***
10 m	−16.559	3.943	171.13	−4.199	4.30 × 10^−5^ ***
15 m	−21.556	3.943	171.13	−5.467	1.60 × 10^−7^ ***
20 m	−19.582	3.943	171.13	−4.966	1.64 × 10^−6^ ***

Notes: The linear mixed-effects model was fitted using restricted maximum likelihood (REML), and *t*-tests were performed using the Satterthwaite approximation for degrees of freedom. Model formula: Germination~Depth + (1|Species). Sample size: 219 observations from 44 species. REML criterion value: 1962.6. Random effects: The variance and standard deviation of the random intercept for Species were 490.9 and 22.16, respectively. Residual variance and standard deviation were 337.3 and 18.37, respectively, indicating substantial variation in baseline germination percentage among species. Significance levels: *** *p* < 0.001. Intercept represents the estimated germination percentage of seeds in the control (0 m depth).

**Table 2:** Results of Bayesian mixed-effects models examining the relationship between seed germination, submergence depth, and seed dormancy traits.

Factors	Predictors	Estimate SE	95% CrI	Rhat	Bulk_ESS	Tail_ESS
Fixed Effects						
Intercept	46.42	14.39	[17.15, 75.03]	1	1943	3318
Submergence depth *	−0.28	3.15	[−6.45, 5.88]	1	5914	5654
Dormancy (Yes vs. No)	2.12	4.95	[−7.53, 11.73]	1	14,334	5812
Dormancy type: Non-dormancy †	−2.08	4.86	[−11.58, 7.37]	1	14,448	6372
Dormancy type: Physical dormancy †	−0.84	4.94	[−10.55, 8.71]	1	12,779	6203
Dormancy type: Physiological dormancy †	−1.13	4.21	[−9.49, 7.10]	1	8870	6377
Dormancy season: None ‡	−2.12	4.87	[−11.60, 7.41]	1	13,641	5773
Dormancy season: Spring-Summer ‡	−1.79	4.61	[−10.84, 7.24]	1	7675	6356
Submergence depth × Dormancy (Yes)	0.48	3.15	[−5.67, 6.60]	1	5911	5594
Submergence depth × Dormancy type: Non-dormancy	−0.82	3.86	[−8.41, 6.53]	1	8393	5762
Submergence depth × Dormancy type: Physical	−1.05	0.9	[−2.81, 0.73]	1	7680	6176
Submergence depth × Dormancy type: Physiological	−0.52	0.4	[−1.31, 0.24]	1	8681	5705
Submergence depth × Dormancy season: None	−0.68	3.82	[−8.06, 6.89]	1	8224	5969
Submergence depth × Dormancy season: Spring/Summer	−1.41	0.38	**[−2.16, −0.67]**	1	8574	6177
**Random Effects**						
Species (Intercept)	35.89	7.97	[21.76, 53.13]	1	1241	2254
Residual standard deviation (σ)	19.81	1.33	[17.49, 22.60]	1	2410	4090

Notes: Model formula: GerminationRate~SubmergenceDepth × (Dormancy + DormancyType + DormancySeason) + (1|gr (Species, cov = phylo_cov)), where phylo_cov represents the phylogenetic covariance matrix. * Submergence depth includes 0 m, 5 m, 10 m, 15 m, and 20 m. † Reference category for dormancy type: Conditional dormancy. ‡ Reference category for dormancy season: Autumn-Winter. Boldfaced 95% CrI indicates credible intervals excluding zero (statistically meaningful effects). Model diagnostics: Rhat ≈ 1 and effective sample sizes (Bulk_ESS, Tail_ESS) > 1000 confirm model convergence and sufficient sampling efficiency. Abbreviations: SE = Standard Error; CrI = Credible Interval; ESS = Effective Sample Size; Rhat = Gelman-Rubin statistic.

**Table 3:** Submergence depth, duration at different elevations in the water level fluctuation zone of the Three Gorges reservoir.

Elevation (m)	Starting Date of Submergence	Ending Date of Submergence	Maximum Submergence Depth (m)	Submergence Duration (Days)
>175	/	/	0	0
170	14 October 2013	21 January 2014	5	100
165	17 September 2013	19 February 2014	10	156
160	12 September 2013	5 May 2014	15	236
155	8 September 2013	15 May 2014	20	250

## Data Availability

The primary data supporting this study were not made publicly available at the time of publication. Data are available from the corresponding author, Bo Zeng, upon request.

## Supplementary Materials

The following supporting information can be downloaded at: https://www.mdpi.com/article/10.3390/plants15111626/s1, Table S1: Fruiting season, seed dormancy status at fruiting, dormancy type, and seed dormancy season of the experimental species, Figure S1: Frequency distribution histograms of seed germination percentages across 44 species under five submergence depths (0 m, 5 m, 10 m, 15 m, and 20 m), and Figure S2: Pagel’s λ (left) and Blomberg’s K (right) of seed germination percentage across species after submergence at different depths.

## Abbreviations

The following abbreviations are used in this manuscript:

TGR	The Three Gorges Reservoir
