# Supplementary material for: Dormancy Season Is Key to Submergence Tolerance of Annual Plant Seeds in the Drawdown Zone of the Three Gorges Reservoir
Source: Plants (Basel). 2026 May 26;15(11):1626. doi: 10.3390/plants15111626 (PMC13258853; doi:10.3390/plants15111626)
Supplement: Supplementary file 1 [file plants-15-01626-s001.zip › plants-4319488-supplementary.pdf]

Table S1. Fruiting season, seed dormancy status at fruiting, dormancy type, and seed dormancy season of the experimental species

Species	Family	Fruiting Period	Dormancy	Dormancy Type	Dormancy Season
Abutilon theophrasti	Malvaceae	Jul-Sep	Yes	Physiological dormancy	Aut-Win
Aeschynomene indica	Leguminosae	Aug-Oct	Yes	Conditional dormancy	Aut-Win
Amaranthus hybridus	Amaranthaceae	Sep-Oct	Yes	Physiological dormancy	Aut-Win
Amaranthus spinosus	Amaranthaceae	Sep-Nov	Yes	Conditional dormancy	Aut-Win
Arthraxon hispidus	Gramineae	Sep-Nov	Yes	Conditional dormancy	Aut-Win
Bidens pilosa	Compositae	Aug-Nov	Yes	Physiological dormancy	Aut-Win
Bidens tripartita	Compositae	Sep-Nov	Yes	Conditional dormancy	Aut-Win
Capsella bursa −pastoris	Cruciferae	Mar-Jun	Yes	Conditional dormancy	Spr-Sum
Celosia argentea	Amaranthaceae	Jun-Oct	Yes	Physiological dormancy	Aut-Win
Cerastium glomeratum	Caryophyllaceae	May-Jun	Yes	Physiological dormancy	Spr-Sum
Chenopodium album	Chenopodiaceae	Mar-Sep	No	None dormancy	None
Chenopodium ambrosioides	Chenopodiaceae	Sep-Oct	Yes	Physiological dormancy	Aut-Win
Cuscuta chinensis	Convolvulaceae	Aug-Oct	Yes	Physical dormancy	Aut-Win

Species	Family	Fruiting Period	Dormancy	Dormancy Type	Dormancy Season
Daucus carota	Umbelliferae	May-Jul	Yes	Physiological dormancy	Spr-Sum
Digitaria sanguinalis	Gramineae	Jun-Sep	Yes	Conditional dormancy	Aut-Win
Echinochloa crusgalli	Gramineae	Jun-Nov	Yes	Physiological dormancy	Aut-Win
Eclipta prostrata	Compositae	Jun-Nov	Yes	Physiological dormancy	Aut-Win
Eleusine indica	Gramineae	Jun-Oct	Yes	Physiological dormancy	Aut-Win
Eriochloa villosa	Gramineae	Jul-Oct	Yes	Physiological dormancy	Aut-Win
Galium trifidum	Rubiaceae	Mar-Jun	Yes	Physiological dormancy	Spr-Sum
Hemistepta lyrata	Compositae	Mar-Aug	Yes	Physiological dormancy	Spr-Sum
Ixeris polycephala	Compositae	Mar-Jun	Yes	Conditional dormancy	Spr-Sum
Leonurus artemisia	Labiatae	Jun-Oct	Yes	Physiological dormancy	Spr-Sum
Leptochloa chinensis	Gramineae	Aug-Nov	Yes	Physiological dormancy	Aut-Win
Medicago lupulina	Leguminosae	May-Jun	Yes	Conditional dormancy	Spr-Sum
Melilotus officinalis	Leguminosae	Jun-Oct	Yes	Physiological dormancy	Spr-Sum
Orychophragmus violaceus	Cruciferae	May-Jun	Yes	Physiological dormancy	Spr-Sum
Plantago asiatica	Plantaginaceae	Jun-Aug	Yes	Physiological dormancy	Spr-Sum

**Species**	**Family**	**Fruiting Period**	**Dormancy**	**Dormancy Type**	**Dormancy Season**
Polygonum lapathifolium	Polygonaceae	Jul-Sep	Yes	Conditional dormancy	Spr-Sum
Polypogon fugax	Gramineae	Apr-Sep	Yes	Physiological dormancy	Spr-Sum
Portulaca oleracea	Portulacaceae	Jun-Sep	Yes	Physiological dormancy	Aut-Win
Ranunculus sceleratus	Ranunculaceae	May-Aug	Yes	Physical dormancy	Spr-Sum
Rorippa indica	Cruciferae	Jun-Aug	Yes	Physiological dormancy	Spr-Sum
Rumex dentatus	Polygonaceae	Jun-Jul	Yes	Physiological dormancy	Spr-Sum
Rumex trisetifer	Polygonaceae	Jun-Jul	Yes	Conditional dormancy	Spr-Sum
Salvi plebeia	Labiatae	Jun-Jul	No	None dormancy	None
Sambucus chinensis	Caprifoliaceae	Jul-Aug	Yes	Conditional dormancy	Spr-Sum
Setaria glauca	Gramineae	Jun-Oct	Yes	Physiological dormancy	Aut-Win
Setaria viridis	Gramineae	May-Oct	Yes	Physiological dormancy	Aut-Win
Solanum nigrum	Solanaceae	Jul-Nov	Yes	Physiological dormancy	Spr-Sum
Stellaria media	Caryophylla-ceae	May-Jul	Yes	Physiological dormancy	Spr-Sum
Torilis scabra	Umbelliferae	May-Jun	Yes	Physiological dormancy	Spr-Sum
Trigonotis peduncularis	Boraginaceae	Mar-Jul	Yes	Physiological dormancy	Spr-Sum

Species	Family	Fruiting Period	Dormancy	Dormancy Type	Dormancy Season
Xanthium sibiricum	Compositae	*Sep-Oct*	Yes	Conditional dormancy	Aut-Win

Note: (i) Seed dormancy for each species was classified at maturity according to the scheme of Baskin & Baskin (2014). (ii) Conditional dormancy-corresponding to the non-deep physiological dormancy in this work, here the use of "conditional dormancy" because this designation applies where partial dormancy attenuation occurred under seasonally fluctuating temperatures without obligatory scarification. (iii) Physiological dormancy- specifically, this type links to the deep physiological dormancy in this work. (iv) Dormancy season (designated as either Spring-Summer or Autumn-Winter) was operationally defined through integrative analysis of seed dormancy status, fruiting phenology, and empirically verified germination characteristics. Categorization relied upon rigorous quantification of phenological synchrony between fruit dispersal phases and germination initiation thresholds under field conditions: seeds possessing confirmed dormancy mechanisms that dispersed during autumn/winter intervals yet exhibited germination primarily in spring/early summer were assigned to the Autumn-Winter class, shorten as "Aut-Win"; reciprocally, dormant seeds dispersing in spring/summer periods with predominant autumn/early winter germination onset comprised the Spring-Summer category, shorten as "Spr-Sum".

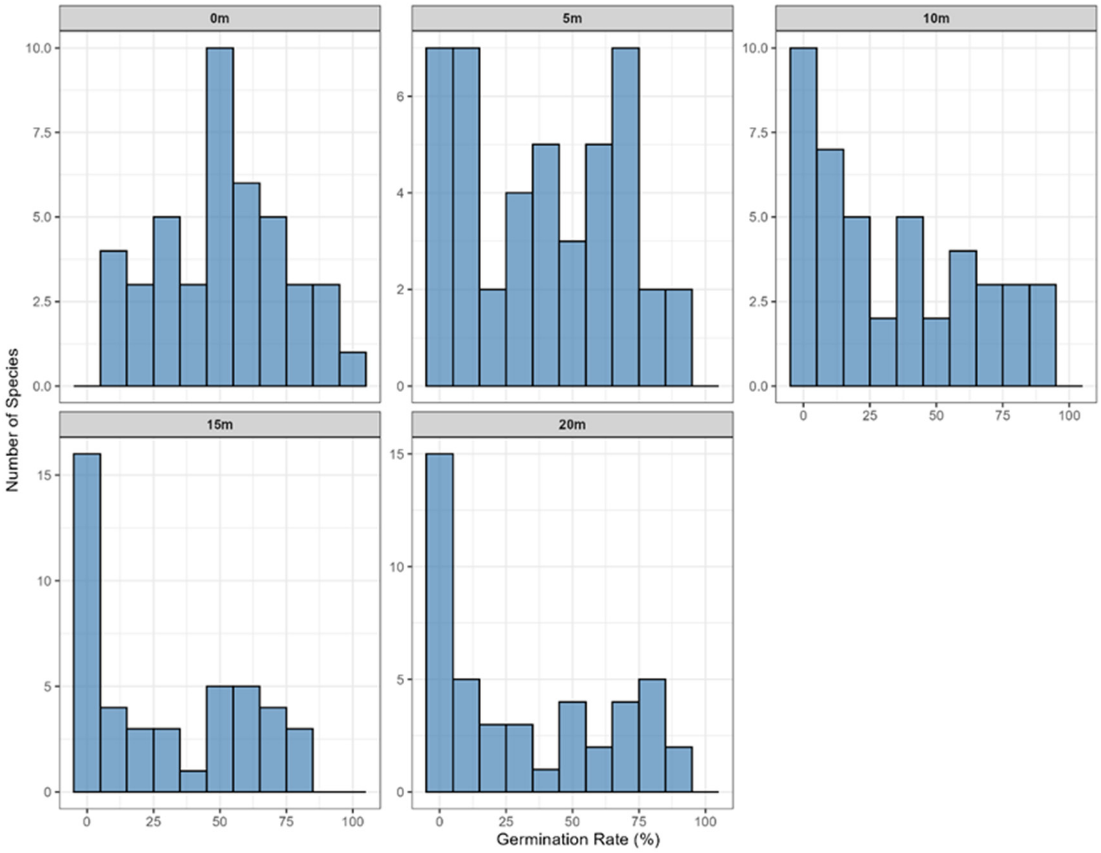

Figure S1. Frequency distribution histograms of seed germination percentages across 44 species under five submergence depths (0 m, 5 m, 10 m, 15 m, and 20 m). Germination percentages represent the mean values from five replicates per species per depth, with each replicate consisting of 200 seeds.

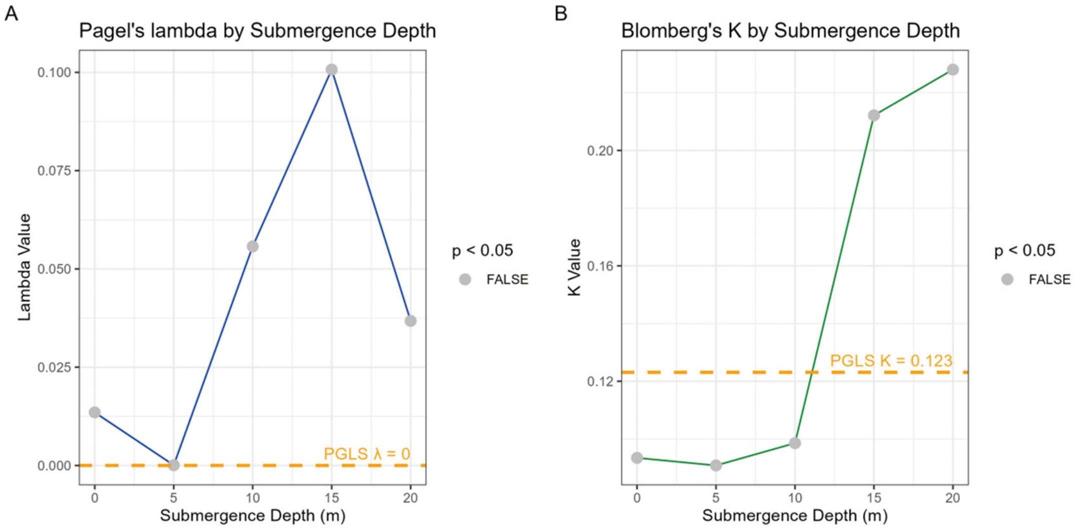

Figure S2. Pagel's λ (left) and Blomberg's K (right) of seed germination percentage across species after submergence at different depths. Solid lines represent the phylogenetic signals of seed germination percentage at different elevations, while horizontal dashed lines represent the overall phylogenetic signals of seed germination percentage (based on PGLS analysis) at different elevations. Values of λ and K were calculated based on 44 species, with germination percentage data obtained after exposure to submergence depths (0 m, 5 m, 10 m, 15 m, 20 m).
